# Crystal structure of a tris(2-amino­eth­yl)methane capped carbamoyl­methyl­phosphine oxide compound

**DOI:** 10.1107/S2056989024008478

**Published:** 2024-08-30

**Authors:** Brandon G. Wackerle, Eric J. Werner, Richard J. Staples, Shannon M. Biros

**Affiliations:** a Department of Chemistry Grand Valley State University,Allendale MI 49401 USA; b Department of Chemistry and Biochemistry The University of Tampa, 401 W Kennedy Blvd Tampa FL 33606 USA; c Center for Crystallographic Research, Department of Chemistry, Michigan State University, East Lansing, MI 48824, USA; University of Buenos Aires, Argentina

**Keywords:** crystal structure, carbamoyl­methyl­phosphine oxide, intra­molecular hydrogen bond, inter­molecular hydrogen bond

## Abstract

The mol­ecular structure of the tripodal carbamoyl­methyl­phosphine oxide compound diethyl {[(5-[2-(di­eth­oxy­phosphor­yl)acetamido]-3-{2-[2-(di­eth­oxy­phos­phor­yl)acetamido]­eth­yl}pent­yl)carbamo­yl]meth­yl}phospho­nate, C_25_H_52_N_3_O_12_P_3_, features six intra­molecular hydrogen-bonding inter­actions. The phospho­nate groups have key bond lengths ranging from 1.4696 (12) to 1.4729 (12) Å (P=O), 1.5681 (11) to 1.5811 (12) Å (P—O) and 1.7881 (16) to 1.7936 (16) Å (P—C). Each amide group adopts a nearly perfect *trans* geometry, and the geometry around each phophorus atom resembles a slightly distorted tetra­hedron.

## Abstract 2

The crystal structure of the compound described here features both C—H⋯O and N—H⋯O intra­molecular hydrogen bonds, as well as a myriad of inter­molecular C—H⋯O hydrogen-bonding inter­actions.

## 1. Chemical context

**Figure 1:**
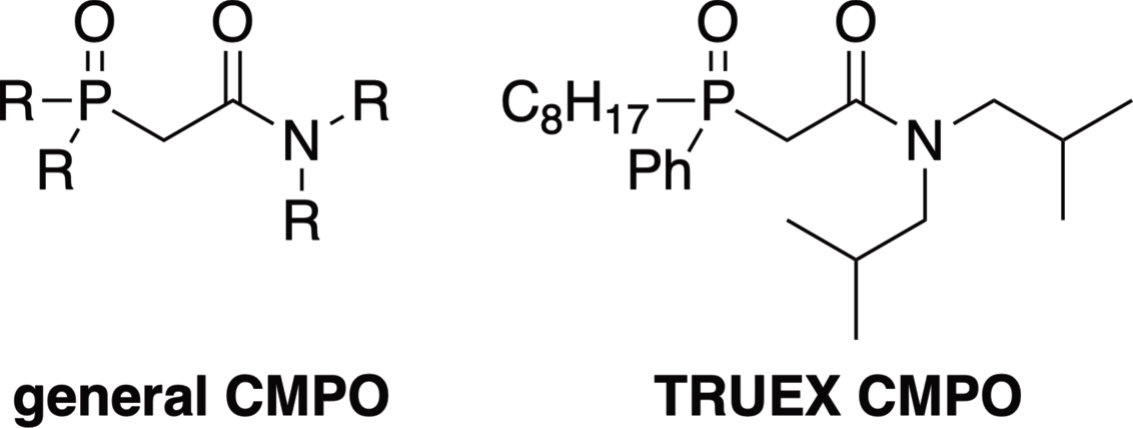
The general structure of the CMPO motif, along with the structure of the CMPO compound used in the TRUEX process.

The carbamoyl­methyl­phosphine oxide (CMPO) group (Fig. 1 ▸) has been utilized by researchers in the area of *f*-element coordination chemistry to prepare compounds with an affinity for lanthanide and actinide metals. Perhaps the most well known use of this metal chelator is as part of the TRUEX (transuranium extraction) process for the remediation of spent nuclear fuel (Horwitz *et al.*, 1985 ▸). Various research groups have studied the coordination complexes of CMPO-containing compounds with *f*-elements and found that, depending on the identity of the metal, two to three CMPO groups are able to coordinate to the metal center simultaneously (Horwitz *et al.*, 1987 ▸). Based on these results, research groups have used a variety of di-, tri- and tetra­podal scaffolds to tether multiple CMPO groups together with the aim of preparing chelators for *f*-elements that have stronger binding affinities and higher extraction selectivities than their monomeric counterparts (Dam *et al.*, 2007 ▸; Leoncini *et al.*, 2017 ▸; Werner & Biros, 2019 ▸). To this end, we have prepared a tripodal CMPO compound based on a *tris*(2-amino­eth­yl)methane scaffold and report here its characterization by X-ray diffraction and NMR spectroscopy.
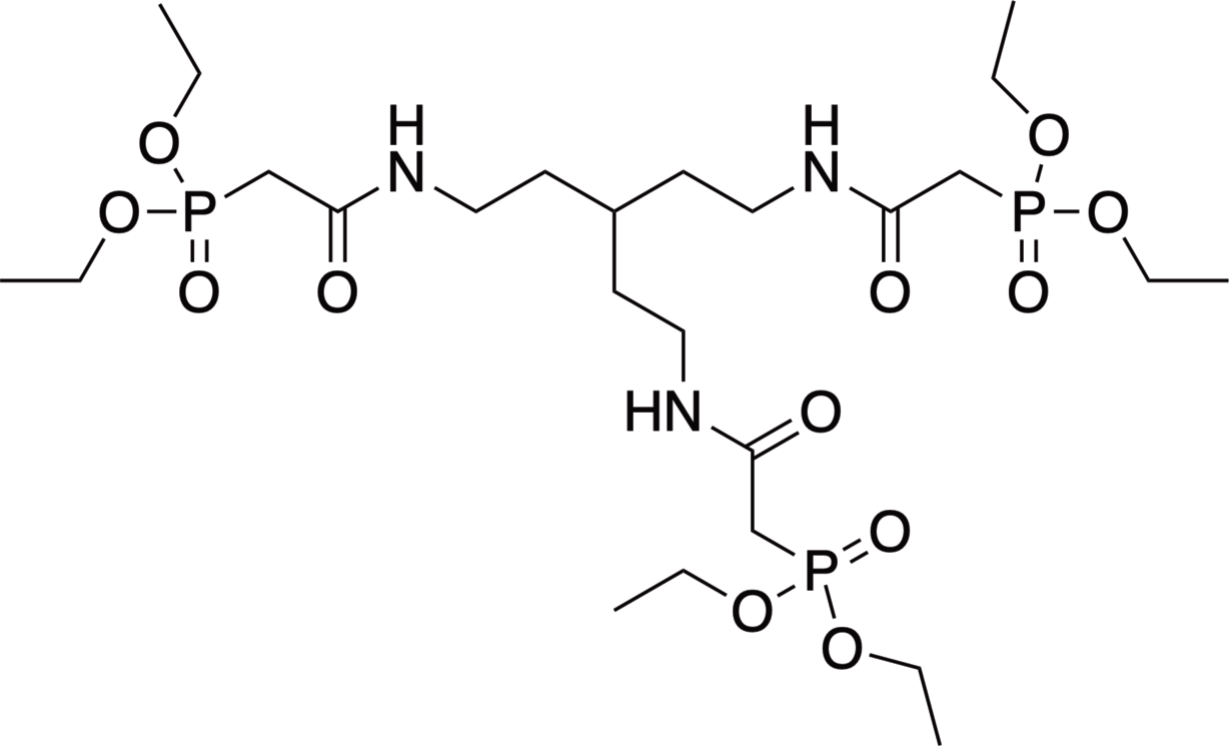


## 2. Structural commentary

**Figure 2:**
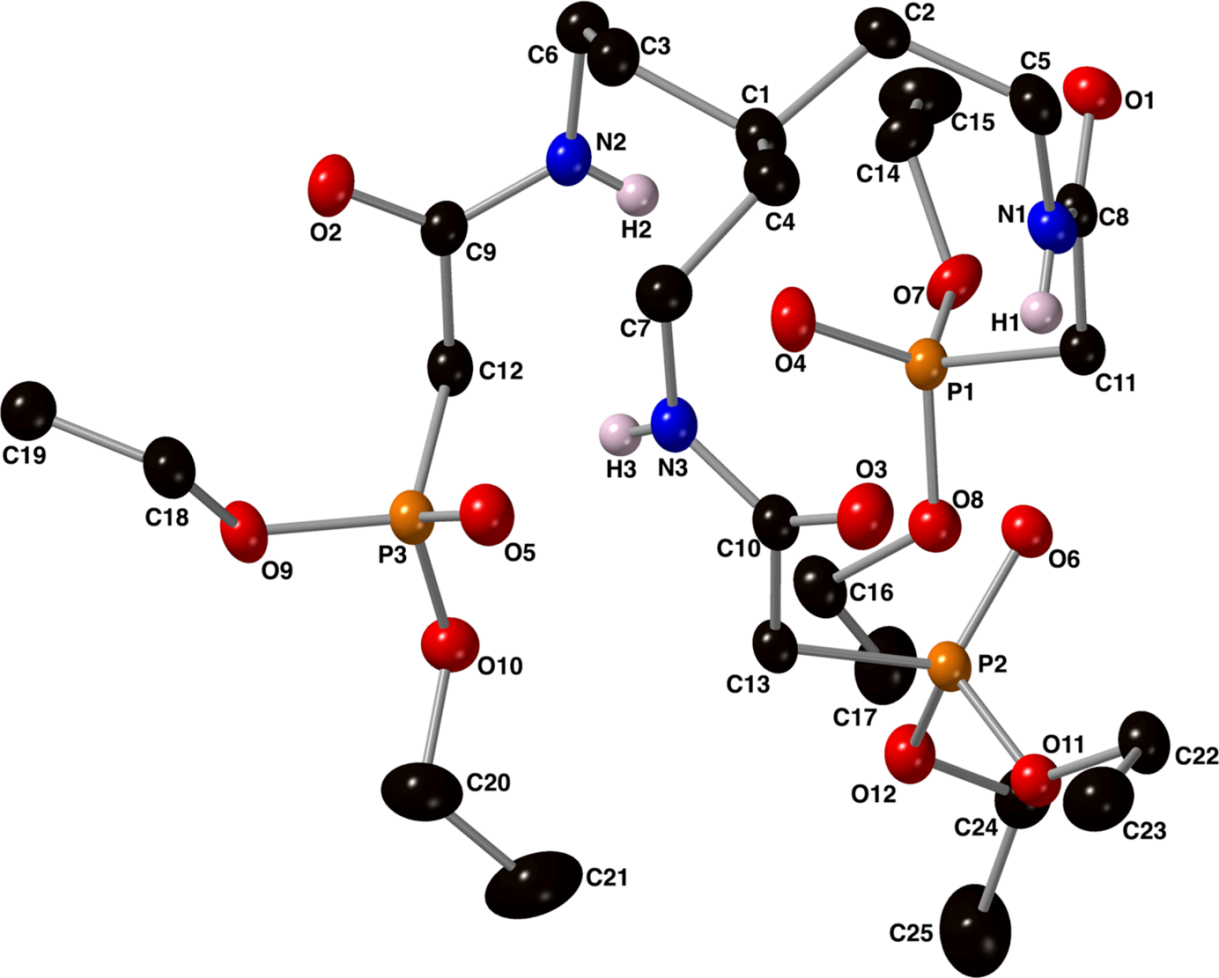
The mol­ecular structure of compound **I**, with the atom-labeling scheme. Displacement ellipsoids are drawn at the 50% probability level, and hydrogen atoms bonded to carbon atoms have been omitted for clarity. With regard to the disordered atoms, only the major component is shown.

The mol­ecular structure of compound **I** is shown in Fig. 2 ▸ along with the atom-numbering scheme. The electron density corresponding to the capping carbon atoms C2, C3 and C4 was disordered and was modeled over two positions with a 0.676 (3):0.324 (3) occupancy ratio (see the *Refinement* section for more details). The three CMPO arms are oriented on the same side of the mol­ecule, and each phopshonate group is engaged in intra­molecular hydrogen bonds with a neighboring amide group (*vide infra*). For the phospho­nate groups, the three P=O bond lengths have values of 1.4696 (12), 1.4722 (12) and 1.4729 (12) Å. The longer P—O bond lengths range from 1.5681 (11) to 1.5811 (12) Å with P—C bond lengths ranging from 1.7881 (16) to 1.7936 (16) Å. Each phospho­rus atom has a τ_4_ descriptor of fourfold coordination of 0.92 (where 0.00 = square planar, 0.85 = trigonal pyramidal, and 1.00 = tetra­hedral; Yang *et al.*, 2007 ▸), indicating that the geometry around these atoms resembles a slightly distorted tetra­hedron. The C=O bond lengths of the amide groups are nearly identical with values of 1.231 (2), 1.231 (2) and 1.230 (2) Å. The C(O)—N bond lengths range from 1.335 (2) to 1.344 (2) Å, and each amide group adopts a nearly perfect *trans* geometry with H—N—C—O torsion angles of 176.9 (19), 177.9 (18) and 179.0 (16)°.

**Figure 3:**
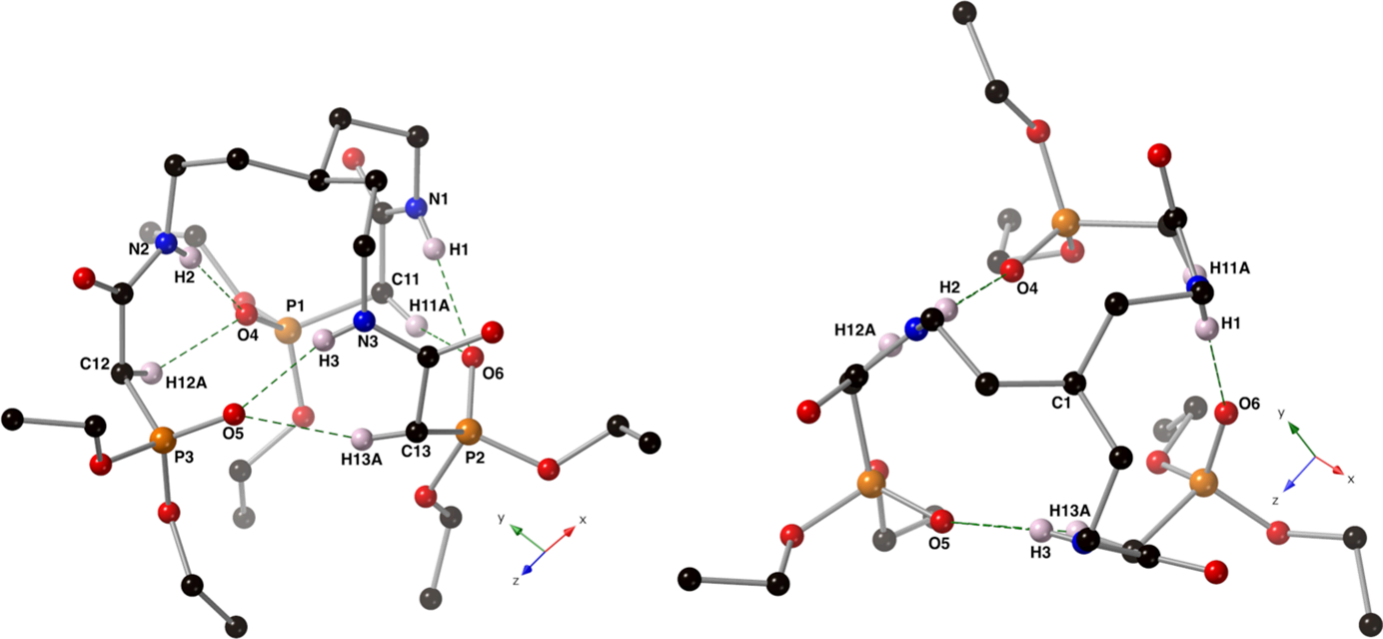
Depictions of the intra­molecular C—H⋯O and N—H⋯O hydrogen bonds (green, dashed lines) present in the crystal of compound **I** using a ball-and-stick model with standard CPK colors. With regard to the disordered atoms, only the major component is shown.

Intra­molecular N—H⋯O and C—H⋯O hydrogen bonds are present in the crystal of compound **I** between each of the P=O oxygen atoms and a neighboring amide group (Fig. 3 ▸ and Table 1 ▸). These inter­actions have an average *D*⋯*A* distance of 2.886 Å and an average *D*—H⋯*A* angle of 169° for the N—H⋯O inter­actions, and an average *D*⋯*A* distance of 3.250 Å and an average *D*—H⋯*A* angle of 148° for the C—H⋯O inter­actions.

## 3. Supra­molecular features

In the crystal, mol­ecules of the title compound form supra­molecular sheets that bis­ect the *y*- and *z*-axes. These sheets are held together by C—H⋯O hydrogen bonds (Table 1 ▸). Additional C—H⋯O hydrogen bonds are found between the supra­molecular sheets.

## 4. Database survey

A search of the Cambridge Structure Database (CSD version 5.44 with updates through June 2024; Groom *et al.*, 2016 ▸) for structures containing the general CMPO motif returned 104 hits, 63 of which were exclusively organic compounds. Of these 63 compounds, 14 structures contained the CMPO moiety tethered to a di-, tri- or tetra­podal scaffold. Structures CIWFAR (Ouizem *et al.*, 2014 ▸) and GOGZAG (VanderWeide *et al.*, 2019 ▸) contain aromatic rings decorated with two CMPO groups. Structures containing three CMPO groups tethered together can be found in entries IMIDEP (Coburn *et al.*, 2016 ▸), XILJOR (Peters *et al.*, 2002 ▸), JIVSUD and JIVTAK (Matloka *et al.*, 2007 ▸). Lastly, a calix[4]arene scaffold was used to link four CMPO groups together in structures OLUWEX (Schmidt *et al.*, 2003 ▸), CUVNEN and CUVNIR (Rudzevich *et al.*, 2010 ▸).

## 5. Synthesis and crystallization

A 25 mL round-bottom flask was charged with 1.15 g (7.90 mmol) of freshly distilled 1,1,1-*tris*(2-amino­eth­yl)methane (Archer *et al.*, 2004 ▸) and 1.0 mL of methanol. Under an atmosphere of nitro­gen, the solution was cooled to *ca*. 230 K with a liquid N_2_/EtOAc bath. Tri­ethyl­phosphono­acetate (6.50 mL, 32.8 mmol) was added slowly to the flask *via* syringe, and the reaction was allowed to warm to room temperature. The reaction was stirred under an inert atmosphere for 3 days, and the volatiles were removed under reduced pressure. The crude product was purified *via* silica gel column chromatography (5–10% MeOH/CH_2_Cl_2_ gradient) to give compound **I** as a slightly yellow, waxy solid (typical yield = 50–60%, *R*_f_ in 10% MeOH/CH_2_Cl_2_ = 0.4). Crystals suitable for analysis by X-ray diffraction were grown serendipitously from a concentrated solution of compound **I** in methanol upon standing in the refrigerator for many months. NMR data was acquired with a JEOL ECZS 400 NMR spectrometer: ^1^H NMR (400 MHz, CDCl_3_) δ 8.24 (broad, 3H), 4.10 (*m*, 12 H), 3.22 (*m*, 6H), 2.95 (*d*, *J*_P–H_ = 21.6 Hz, 6H), 1.68 (septet, *J* = 6.8 Hz, 3H), 1.40 (*dt*, *J* = 6.3, 13.7 Hz, 6H), 1.29 (*t*, *J* = 7.1 Hz, 18H); ^13^C NMR (100 MHz, CDCl_3_) δ 164.8 (*d*, *J*_C–P_ = 5.2 Hz), 62.5 (*d*, *J*_C-P_ = 6.4 Hz), 36.3 (*s*), 35.0 (*d*, *J*_C–P_ = 132 Hz), 31.8 (*s*), 25.2 (*s*), 16.4 (*d*, *J*_C–P_ = 6.3 Hz); ^31^P NMR (161 MHz, CDCl_3_) δ 24.4.

## 6. Refinement

Crystal data, data collection and structure refinement details are summarized in Table 2 ▸. All hydrogen atoms bonded to carbon atoms were placed in calculated positions and refined as riding: C—H = 0.95–1.00 Å with *U*_iso_(H) = 1.2*U*_eq_(C) for methyl­ene and methine groups, and *U*_iso_(H) = 1.5*U*_eq_(C) for methyl groups. Hydrogen atoms bonded to nitro­gen atoms were located using electron-density difference maps. The disordered electron density corresponding to C2/C2*A*, C3/C3*A* and C4/C4*A* was modeled over two positions and refined against a free variable to give a relative occupancy ratio of 0.676 (3):0.324 (3). This disorder reverberated to the nearby carbon atoms C5, C6 and C7 to give two orientations of the attached hydrogen atoms.

## Supplementary Material

Crystal structure: contains datablock(s) I. DOI: 10.1107/S2056989024008478/vu2007sup1.cif

Structure factors: contains datablock(s) I. DOI: 10.1107/S2056989024008478/vu2007Isup3.hkl

Supporting information file. DOI: 10.1107/S2056989024008478/vu2007Isup3.cml

CCDC reference: 2379899

Additional supporting information:  crystallographic information; 3D view; checkCIF report

## Figures and Tables

**Table 1:** Hydrogen-bond geometry (Å, °)

*D*—H⋯*A*	*D*—H	H⋯*A*	*D*⋯*A*	*D*—H⋯*A*
N1—H1⋯O6	0.86 (2)	2.07 (2)	2.9138 (18)	168.8 (19)
N2—H2⋯O4	0.79 (2)	2.06 (2)	2.8465 (18)	170 (2)
N3—H3⋯O5	0.82 (2)	2.10 (2)	2.8975 (19)	167 (2)
C11—H11*A*⋯O6	0.99	2.36	3.2433 (19)	148
C11—H11*B*⋯O6^i^	0.99	2.48	3.3235 (19)	143
C12—H12*A*⋯O4	0.99	2.37	3.2476 (19)	148
C12—H12*B*⋯O2^ii^	0.99	2.35	3.321 (2)	168
C13—H13*A*⋯O5	0.99	2.37	3.259 (2)	149
C14—H14*A*⋯O1	0.99	2.56	3.326 (2)	135
C17—H17*B*⋯O3^iii^	0.98	2.65	3.427 (3)	137
C18—H18*A*⋯O2	0.99	2.57	3.215 (2)	122
C22—H22*B*⋯O1^i^	0.99	2.80	3.472 (2)	126
C23—H23*C*⋯O3	0.98	2.69	3.460 (2)	135
C24—H24*A*⋯O1^i^	0.99	2.55	3.480 (2)	156
C24—H24*B*⋯O8	0.99	2.57	3.444 (2)	147
C4*A*—H4*AA*⋯O2^iv^	0.99	2.39	3.241 (5)	144

Symmetry codes: (i) 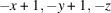; (ii) 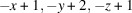; (iii) 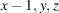; (iv) 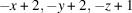.

**Table 2:** Experimental details

Crystal data	
Chemical formula	C_25_H_52_N_3_O_12_P_3_
*M*_r_	679.60
Crystal system, space group	Triclinic, *P* 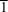
Temperature (K)	100
*a*, *b*, *c* (Å)	10.02487 (11), 11.92992 (15), 16.6237 (2)
α, β, γ (°)	100.4792 (11), 100.124 (1), 111.1313 (11)
*V* (Å^3^)	1759.25 (4)
*Z*	2
Radiation type	Cu *K*α
μ (mm^−1^)	2.06
Crystal size (mm)	0.16 × 0.09 × 0.04

Data collection	
Diffractometer	XtaLAB Synergy-S, Dualflex, HyPix-6000HE
Absorption correction	Gaussian (*CrysAlis PRO*; Oxford Diffraction, 2006 ▸)
*T*_min_, *T*_max_	0.700, 1.000
No. of measured, independent and observed [*I* > 2σ(*I*)] reflections	27502, 7504, 6481
*R*_int_	0.043
(sin θ/λ)_max_ (Å^−1^)	0.639

Refinement	
*R*[*F*^2^ > 2σ(*F*^2^)], *wR*(*F*^2^), *S*	0.037, 0.099, 1.07
No. of reflections	7504
No. of parameters	438
H-atom treatment	H atoms treated by a mixture of independent and constrained refinement
Δρ_max_, Δρ_min_ (e Å^−3^)	0.61, −0.35

Computer programs: *CrysAlis PRO* (Oxford Diffraction, 2006 ▸), *SHELXT* (Sheldrick, 2015*a* ▸), *SHELXL2019/2* (Sheldrick, 2015*b* ▸), *CrystalMaker* (Palmer, 2007 ▸) and *OLEX2* (Dolomanov *et al.*, 2009 ▸; Bourhis *et al.*, 2015 ▸).

## supplementary crystallographic information

### Diethyl {[(5-[2-(diethoxyphosphoryl)acetamido]-3-{2-[2-(diethoxyphosphoryl)acetamido]ethyl}pentyl)carbamoyl]methyl}phosphonate Crystal data

**Table:**

C_25_H_52_N_3_O_12_P_3_	*Z* = 2
*M**_r_* = 679.60	*F*(000) = 728
Triclinic, *P*1	*D*_x_ = 1.283 Mg m^−^^3^
*a* = 10.02487 (11) Å	Cu *K*α radiation, λ = 1.54184 Å
*b* = 11.92992 (15) Å	Cell parameters from 13811 reflections
*c* = 16.6237 (2) Å	θ = 4.1–79.9°
α = 100.4792 (11)°	µ = 2.06 mm^−^^1^
β = 100.124 (1)°	*T* = 100 K
γ = 111.1313 (11)°	Irregular, colourless
*V* = 1759.25 (4) Å^3^	0.16 × 0.09 × 0.04 mm

### Diethyl {[(5-[2-(diethoxyphosphoryl)acetamido]-3-{2-[2-(diethoxyphosphoryl)acetamido]ethyl}pentyl)carbamoyl]methyl}phosphonate Data collection

**Table:**

XtaLAB Synergy-S, Dualflex, HyPix-6000HE diffractometer	6481 reflections with *I* > 2σ(*I*)
Detector resolution: 10.0000 pixels mm^-1^	*R*_int_ = 0.043
ω scans	θ_max_ = 80.2°, θ_min_ = 2.8°
Absorption correction: gaussian (CrysAlisPro; Oxford Diffraction, 2006)	*h* = −12→12
*T*_min_ = 0.700, *T*_max_ = 1.000	*k* = −15→14
27502 measured reflections	*l* = −20→21
7504 independent reflections	

### Diethyl {[(5-[2-(diethoxyphosphoryl)acetamido]-3-{2-[2-(diethoxyphosphoryl)acetamido]ethyl}pentyl)carbamoyl]methyl}phosphonate Refinement

**Table:**

Refinement on *F*^2^	Primary atom site location: dual
Least-squares matrix: full	Secondary atom site location: difference Fourier map
*R*[*F*^2^ > 2σ(*F*^2^)] = 0.037	Hydrogen site location: mixed
*wR*(*F*^2^) = 0.099	H atoms treated by a mixture of independent and constrained refinement
*S* = 1.07	*w* = 1/[σ^2^(*F*_o_^2^) + (0.0443*P*)^2^ + 0.5632*P*] where *P* = (*F*_o_^2^ + 2*F*_c_^2^)/3
7504 reflections	(Δ/σ)_max_ = 0.001
438 parameters	Δρ_max_ = 0.61 e Å^−^^3^
0 restraints	Δρ_min_ = −0.34 e Å^−^^3^

### Diethyl {[(5-[2-(diethoxyphosphoryl)acetamido]-3-{2-[2-(diethoxyphosphoryl)acetamido]ethyl}pentyl)carbamoyl]methyl}phosphonate Special details

**Table:**

Geometry. All esds (except the esd in the dihedral angle between two l.s. planes) are estimated using the full covariance matrix. The cell esds are taken into account individually in the estimation of esds in distances, angles and torsion angles; correlations between esds in cell parameters are only used when they are defined by crystal symmetry. An approximate (isotropic) treatment of cell esds is used for estimating esds involving l.s. planes.

### Diethyl {[(5-[2-(diethoxyphosphoryl)acetamido]-3-{2-[2-(diethoxyphosphoryl)acetamido]ethyl}pentyl)carbamoyl]methyl}phosphonate Fractional atomic coordinates and isotropic or equivalent isotropic displacement parameters (Å^2^)

**Table:**

	*x*	*y*	*z*	*U*_iso_*/*U*_eq_	Occ. (<1)
P1	0.35890 (4)	0.68848 (4)	0.12527 (2)	0.02154 (10)	
P2	0.53282 (4)	0.38100 (4)	0.20227 (2)	0.02071 (9)	
P3	0.47290 (4)	0.71266 (4)	0.45959 (2)	0.02314 (10)	
O1	0.62953 (12)	0.82490 (11)	0.04850 (8)	0.0286 (2)	
O2	0.69588 (13)	0.99779 (11)	0.49775 (7)	0.0314 (3)	
O3	0.86359 (13)	0.48663 (12)	0.29651 (8)	0.0336 (3)	
O4	0.45143 (13)	0.75898 (12)	0.21164 (7)	0.0310 (3)	
O5	0.58783 (13)	0.66867 (11)	0.44337 (8)	0.0304 (3)	
O6	0.59114 (12)	0.46394 (10)	0.14955 (7)	0.0261 (2)	
O7	0.28427 (12)	0.76460 (10)	0.07967 (8)	0.0283 (2)	
O8	0.22248 (12)	0.56567 (11)	0.11756 (8)	0.0276 (2)	
O9	0.48562 (12)	0.75646 (11)	0.55729 (7)	0.0274 (2)	
O10	0.30802 (13)	0.61449 (11)	0.42340 (8)	0.0299 (3)	
O11	0.53576 (12)	0.24858 (10)	0.17424 (7)	0.0249 (2)	
O12	0.36511 (12)	0.34604 (11)	0.20133 (7)	0.0267 (2)	
N1	0.71852 (14)	0.70410 (14)	0.11270 (9)	0.0242 (3)	
H1	0.693 (2)	0.636 (2)	0.1277 (13)	0.025 (5)*	
N2	0.66717 (15)	0.94335 (13)	0.35606 (9)	0.0248 (3)	
H2	0.614 (2)	0.896 (2)	0.3124 (15)	0.030 (5)*	
N3	0.82763 (15)	0.64181 (13)	0.37830 (9)	0.0250 (3)	
H3	0.766 (2)	0.661 (2)	0.3961 (14)	0.029 (5)*	
C1	0.89282 (17)	0.85561 (16)	0.29672 (10)	0.0270 (3)	
H1A	0.791 (2)	0.7973 (18)	0.2865 (13)	0.025 (5)*	
C5	0.87290 (17)	0.79247 (17)	0.13561 (11)	0.0296 (3)	
H5AA	0.893030	0.827828	0.087376	0.036*	0.676 (3)
H5AB	0.937439	0.747536	0.145957	0.036*	0.676 (3)
H5BC	0.878553	0.876708	0.134409	0.036*	0.324 (3)
H5BD	0.924175	0.767149	0.094618	0.036*	0.324 (3)
C6	0.81521 (18)	1.02970 (15)	0.35924 (11)	0.0282 (3)	
H6AA	0.846342	1.105489	0.406285	0.034*	0.676 (3)
H6AB	0.812996	1.055031	0.305686	0.034*	0.676 (3)
H6BC	0.882153	1.049504	0.416062	0.034*	0.324 (3)
H6BD	0.811939	1.108309	0.349705	0.034*	0.324 (3)
C7	0.97830 (17)	0.73567 (16)	0.39527 (11)	0.0287 (3)	
H7AA	1.049062	0.696112	0.404807	0.034*	0.676 (3)
H7AB	0.999863	0.800928	0.447785	0.034*	0.676 (3)
H7BC	1.030570	0.707191	0.356172	0.034*	0.324 (3)
H7BD	1.034145	0.749897	0.454197	0.034*	0.324 (3)
C8	0.60896 (16)	0.72964 (15)	0.07237 (9)	0.0223 (3)	
C9	0.62078 (18)	0.93312 (14)	0.42602 (10)	0.0243 (3)	
C10	0.78257 (17)	0.52657 (15)	0.32878 (10)	0.0249 (3)	
C11	0.45376 (16)	0.63076 (14)	0.05793 (10)	0.0223 (3)	
H11A	0.459884	0.554470	0.070441	0.027*	
H11B	0.398214	0.608946	−0.002128	0.027*	
C12	0.46608 (17)	0.83407 (15)	0.41182 (10)	0.0249 (3)	
H12A	0.419042	0.798180	0.350200	0.030*	
H12B	0.404881	0.872627	0.436542	0.030*	
C13	0.61943 (17)	0.44487 (15)	0.31384 (10)	0.0258 (3)	
H13A	0.569838	0.494936	0.339364	0.031*	
H13B	0.609095	0.376363	0.341386	0.031*	
C14	0.36017 (19)	0.89977 (15)	0.09918 (12)	0.0308 (4)	
H14A	0.447502	0.922709	0.075691	0.037*	
H14B	0.394467	0.936564	0.161344	0.037*	
C15	0.2526 (2)	0.94742 (19)	0.06022 (16)	0.0436 (5)	
H15A	0.217905	0.909149	−0.001106	0.065*	
H15B	0.301497	1.038212	0.071238	0.065*	
H15C	0.167935	0.926141	0.085007	0.065*	
C16	0.1174 (2)	0.56953 (19)	0.16770 (12)	0.0359 (4)	
H16A	0.136218	0.657160	0.193669	0.043*	
H16B	0.129442	0.528868	0.213731	0.043*	
C17	−0.0359 (2)	0.5030 (2)	0.11089 (15)	0.0528 (6)	
H17A	−0.046762	0.543631	0.065403	0.079*	
H17B	−0.107391	0.505756	0.143687	0.079*	
H17C	−0.054202	0.416041	0.086221	0.079*	
C18	0.62943 (17)	0.81483 (17)	0.61951 (10)	0.0287 (3)	
H18A	0.708701	0.850973	0.591946	0.034*	
H18B	0.650082	0.752204	0.645208	0.034*	
C19	0.6248 (2)	0.91549 (17)	0.68647 (12)	0.0343 (4)	
H19A	0.612075	0.980235	0.661174	0.051*	
H19B	0.717792	0.952208	0.731421	0.051*	
H19C	0.541637	0.879707	0.710529	0.051*	
C20	0.2548 (2)	0.5026 (2)	0.45184 (15)	0.0457 (5)	
H20A	0.209606	0.517026	0.498961	0.055*	
H20B	0.338692	0.481182	0.472970	0.055*	
C21	0.1447 (3)	0.3995 (2)	0.38158 (18)	0.0587 (7)	
H21A	0.191132	0.382672	0.336272	0.088*	
H21B	0.063215	0.422058	0.359740	0.088*	
H21C	0.105937	0.324849	0.401657	0.088*	
C22	0.64205 (19)	0.22577 (17)	0.13194 (11)	0.0291 (3)	
H22A	0.708598	0.304258	0.122922	0.035*	
H22B	0.589477	0.164576	0.075948	0.035*	
C23	0.7309 (2)	0.17610 (18)	0.18627 (13)	0.0364 (4)	
H23A	0.797129	0.153545	0.156173	0.055*	
H23B	0.663732	0.102142	0.198646	0.055*	
H23C	0.789814	0.240173	0.239481	0.055*	
C24	0.25179 (18)	0.28757 (16)	0.12104 (11)	0.0298 (3)	
H24A	0.282073	0.234942	0.081284	0.036*	
H24B	0.237004	0.352005	0.095038	0.036*	
C25	0.1120 (2)	0.2097 (3)	0.13888 (17)	0.0668 (8)	
H25A	0.032790	0.170273	0.085977	0.100*	
H25B	0.084140	0.262460	0.179047	0.100*	
H25C	0.127335	0.145065	0.163357	0.100*	
C2	0.9138 (3)	0.9024 (2)	0.21687 (16)	0.0273 (5)	0.676 (3)
H2A	1.018299	0.961080	0.227461	0.033*	0.676 (3)
H2B	0.850660	0.948454	0.206276	0.033*	0.676 (3)
C3	0.9259 (3)	0.9732 (2)	0.37186 (16)	0.0286 (6)	0.676 (3)
H3A	0.927444	0.947845	0.425396	0.034*	0.676 (3)
H3B	1.025648	1.037297	0.377840	0.034*	0.676 (3)
C4	1.0018 (2)	0.7972 (2)	0.32176 (16)	0.0276 (6)	0.676 (3)
H4A	1.103868	0.862754	0.338242	0.033*	0.676 (3)
H4B	0.993196	0.733465	0.271490	0.033*	0.676 (3)
C2A	0.9477 (5)	0.7944 (5)	0.2254 (3)	0.0269 (11)	0.324 (3)
H2AA	1.056167	0.840578	0.236832	0.032*	0.324 (3)
H2AB	0.928389	0.707658	0.227536	0.032*	0.324 (3)
C3A	0.8789 (5)	0.9696 (5)	0.2877 (3)	0.0263 (11)	0.324 (3)
H3AA	0.812478	0.952468	0.231216	0.032*	0.324 (3)
H3AB	0.977608	1.031831	0.289758	0.032*	0.324 (3)
C4A	0.9682 (6)	0.8531 (5)	0.3825 (3)	0.0292 (12)	0.324 (3)
H4AA	1.070549	0.918112	0.398768	0.035*	0.324 (3)
H4AB	0.916861	0.878735	0.423347	0.035*	0.324 (3)

### Diethyl {[(5-[2-(diethoxyphosphoryl)acetamido]-3-{2-[2-(diethoxyphosphoryl)acetamido]ethyl}pentyl)carbamoyl]methyl}phosphonate Atomic displacement parameters (Å^2^)

**Table:**

	*U*^11^	*U*^22^	*U*^33^	*U*^12^	*U*^13^	*U*^23^
P1	0.01846 (17)	0.02538 (19)	0.01788 (19)	0.00752 (15)	0.00438 (14)	0.00221 (14)
P2	0.01955 (18)	0.02467 (19)	0.01902 (18)	0.00989 (14)	0.00611 (13)	0.00554 (14)
P3	0.02313 (19)	0.02747 (19)	0.01929 (19)	0.01144 (15)	0.00539 (14)	0.00510 (14)
O1	0.0275 (6)	0.0315 (6)	0.0313 (6)	0.0127 (5)	0.0109 (5)	0.0142 (5)
O2	0.0338 (6)	0.0328 (6)	0.0204 (6)	0.0105 (5)	0.0035 (5)	−0.0005 (5)
O3	0.0279 (6)	0.0382 (7)	0.0339 (7)	0.0157 (5)	0.0088 (5)	0.0023 (5)
O4	0.0263 (5)	0.0411 (7)	0.0188 (6)	0.0095 (5)	0.0052 (4)	0.0013 (5)
O5	0.0339 (6)	0.0344 (6)	0.0303 (6)	0.0201 (5)	0.0119 (5)	0.0095 (5)
O6	0.0278 (5)	0.0287 (6)	0.0236 (6)	0.0116 (4)	0.0083 (4)	0.0098 (4)
O7	0.0268 (5)	0.0222 (5)	0.0293 (6)	0.0099 (4)	0.0001 (4)	−0.0014 (4)
O8	0.0227 (5)	0.0292 (6)	0.0307 (6)	0.0088 (4)	0.0128 (5)	0.0056 (5)
O9	0.0212 (5)	0.0406 (6)	0.0191 (5)	0.0114 (5)	0.0051 (4)	0.0074 (5)
O10	0.0274 (6)	0.0306 (6)	0.0272 (6)	0.0084 (5)	0.0021 (5)	0.0092 (5)
O11	0.0248 (5)	0.0267 (5)	0.0265 (6)	0.0124 (4)	0.0106 (4)	0.0072 (4)
O12	0.0210 (5)	0.0334 (6)	0.0258 (6)	0.0122 (4)	0.0069 (4)	0.0046 (5)
N1	0.0208 (6)	0.0309 (7)	0.0218 (6)	0.0101 (5)	0.0067 (5)	0.0090 (5)
N2	0.0257 (6)	0.0264 (7)	0.0185 (6)	0.0089 (5)	0.0036 (5)	0.0026 (5)
N3	0.0223 (6)	0.0299 (7)	0.0205 (6)	0.0094 (5)	0.0056 (5)	0.0043 (5)
C1	0.0221 (7)	0.0363 (9)	0.0229 (8)	0.0120 (7)	0.0064 (6)	0.0082 (6)
C5	0.0193 (7)	0.0441 (9)	0.0247 (8)	0.0098 (7)	0.0094 (6)	0.0102 (7)
C6	0.0287 (8)	0.0259 (8)	0.0253 (8)	0.0074 (6)	0.0054 (6)	0.0050 (6)
C7	0.0219 (7)	0.0327 (8)	0.0254 (8)	0.0080 (6)	−0.0002 (6)	0.0063 (6)
C8	0.0217 (7)	0.0294 (7)	0.0168 (7)	0.0107 (6)	0.0076 (5)	0.0058 (6)
C9	0.0284 (7)	0.0246 (7)	0.0214 (7)	0.0140 (6)	0.0057 (6)	0.0040 (6)
C10	0.0250 (7)	0.0307 (8)	0.0188 (7)	0.0116 (6)	0.0046 (6)	0.0068 (6)
C11	0.0208 (7)	0.0262 (7)	0.0198 (7)	0.0097 (6)	0.0056 (5)	0.0050 (6)
C12	0.0273 (7)	0.0291 (8)	0.0202 (7)	0.0143 (6)	0.0066 (6)	0.0047 (6)
C13	0.0269 (7)	0.0293 (8)	0.0196 (7)	0.0090 (6)	0.0083 (6)	0.0058 (6)
C14	0.0298 (8)	0.0217 (7)	0.0369 (9)	0.0092 (6)	0.0091 (7)	0.0008 (6)
C15	0.0374 (10)	0.0335 (9)	0.0681 (14)	0.0189 (8)	0.0184 (9)	0.0191 (9)
C16	0.0316 (9)	0.0430 (10)	0.0327 (9)	0.0110 (8)	0.0190 (7)	0.0078 (8)
C17	0.0273 (9)	0.0609 (13)	0.0509 (13)	0.0022 (9)	0.0211 (9)	−0.0080 (10)
C18	0.0213 (7)	0.0405 (9)	0.0211 (8)	0.0107 (7)	0.0019 (6)	0.0082 (7)
C19	0.0344 (9)	0.0334 (9)	0.0278 (9)	0.0085 (7)	0.0051 (7)	0.0051 (7)
C20	0.0384 (10)	0.0418 (11)	0.0491 (12)	0.0078 (8)	−0.0002 (9)	0.0229 (9)
C21	0.0462 (12)	0.0369 (11)	0.0729 (17)	0.0088 (9)	−0.0149 (11)	0.0139 (11)
C22	0.0301 (8)	0.0363 (9)	0.0292 (8)	0.0201 (7)	0.0137 (7)	0.0086 (7)
C23	0.0276 (8)	0.0367 (9)	0.0468 (11)	0.0175 (7)	0.0056 (7)	0.0101 (8)
C24	0.0239 (7)	0.0346 (8)	0.0293 (8)	0.0143 (7)	0.0017 (6)	0.0047 (7)
C25	0.0233 (9)	0.094 (2)	0.0500 (14)	−0.0012 (11)	0.0086 (9)	−0.0044 (13)
C2	0.0212 (11)	0.0321 (12)	0.0270 (12)	0.0070 (9)	0.0073 (9)	0.0113 (10)
C3	0.0223 (11)	0.0332 (12)	0.0235 (12)	0.0072 (9)	0.0019 (9)	0.0038 (10)
C4	0.0183 (10)	0.0355 (13)	0.0273 (12)	0.0089 (9)	0.0056 (9)	0.0091 (10)
C2A	0.019 (2)	0.030 (2)	0.027 (3)	0.0078 (19)	0.0054 (18)	0.0021 (19)
C3A	0.025 (2)	0.025 (2)	0.025 (2)	0.0073 (19)	0.0072 (19)	0.0052 (19)
C4A	0.026 (2)	0.029 (2)	0.024 (3)	0.007 (2)	0.0009 (19)	0.0017 (19)

### Diethyl {[(5-[2-(diethoxyphosphoryl)acetamido]-3-{2-[2-(diethoxyphosphoryl)acetamido]ethyl}pentyl)carbamoyl]methyl}phosphonate Geometric parameters (Å, º)

**Table:**

P1—O4	1.4696 (12)	C9—C12	1.519 (2)
P1—O7	1.5811 (12)	C10—C13	1.519 (2)
P1—O8	1.5681 (11)	C11—H11A	0.9900
P1—C11	1.7881 (16)	C11—H11B	0.9900
P2—O6	1.4722 (12)	C12—H12A	0.9900
P2—O11	1.5761 (12)	C12—H12B	0.9900
P2—O12	1.5759 (11)	C13—H13A	0.9900
P2—C13	1.7936 (16)	C13—H13B	0.9900
P3—O5	1.4729 (12)	C14—H14A	0.9900
P3—O9	1.5803 (12)	C14—H14B	0.9900
P3—O10	1.5697 (12)	C14—C15	1.495 (3)
P3—C12	1.7899 (17)	C15—H15A	0.9800
O1—C8	1.231 (2)	C15—H15B	0.9800
O2—C9	1.231 (2)	C15—H15C	0.9800
O3—C10	1.230 (2)	C16—H16A	0.9900
O7—C14	1.457 (2)	C16—H16B	0.9900
O8—C16	1.4612 (19)	C16—C17	1.490 (3)
O9—C18	1.4571 (18)	C17—H17A	0.9800
O10—C20	1.448 (2)	C17—H17B	0.9800
O11—C22	1.4515 (19)	C17—H17C	0.9800
O12—C24	1.455 (2)	C18—H18A	0.9900
N1—H1	0.86 (2)	C18—H18B	0.9900
N1—C5	1.457 (2)	C18—C19	1.502 (3)
N1—C8	1.344 (2)	C19—H19A	0.9800
N2—H2	0.79 (2)	C19—H19B	0.9800
N2—C6	1.455 (2)	C19—H19C	0.9800
N2—C9	1.336 (2)	C20—H20A	0.9900
N3—H3	0.82 (2)	C20—H20B	0.9900
N3—C7	1.460 (2)	C20—C21	1.463 (3)
N3—C10	1.335 (2)	C21—H21A	0.9800
C1—H1A	0.97 (2)	C21—H21B	0.9800
C1—C2	1.550 (3)	C21—H21C	0.9800
C1—C3	1.587 (3)	C22—H22A	0.9900
C1—C4	1.532 (3)	C22—H22B	0.9900
C1—C2A	1.564 (5)	C22—C23	1.501 (2)
C1—C3A	1.447 (5)	C23—H23A	0.9800
C1—C4A	1.503 (5)	C23—H23B	0.9800
C5—H5AA	0.9900	C23—H23C	0.9800
C5—H5AB	0.9900	C24—H24A	0.9900
C5—H5BC	0.9900	C24—H24B	0.9900
C5—H5BD	0.9900	C24—C25	1.491 (3)
C5—C2	1.577 (3)	C25—H25A	0.9800
C5—C2A	1.540 (5)	C25—H25B	0.9800
C6—H6AA	0.9900	C25—H25C	0.9800
C6—H6AB	0.9900	C2—H2A	0.9900
C6—H6BC	0.9900	C2—H2B	0.9900
C6—H6BD	0.9900	C3—H3A	0.9900
C6—C3	1.497 (3)	C3—H3B	0.9900
C6—C3A	1.619 (5)	C4—H4A	0.9900
C7—H7AA	0.9900	C4—H4B	0.9900
C7—H7AB	0.9900	C2A—H2AA	0.9900
C7—H7BC	0.9900	C2A—H2AB	0.9900
C7—H7BD	0.9900	C3A—H3AA	0.9900
C7—C4	1.546 (3)	C3A—H3AB	0.9900
C7—C4A	1.490 (6)	C4A—H4AA	0.9900
C8—C11	1.521 (2)	C4A—H4AB	0.9900
			
O4—P1—O7	112.90 (7)	O7—C14—H14A	110.2
O4—P1—O8	116.05 (7)	O7—C14—H14B	110.2
O4—P1—C11	114.00 (7)	O7—C14—C15	107.68 (14)
O7—P1—C11	108.90 (7)	H14A—C14—H14B	108.5
O8—P1—O7	102.52 (6)	C15—C14—H14A	110.2
O8—P1—C11	101.26 (7)	C15—C14—H14B	110.2
O6—P2—O11	113.64 (6)	C14—C15—H15A	109.5
O6—P2—O12	115.95 (7)	C14—C15—H15B	109.5
O6—P2—C13	114.70 (7)	C14—C15—H15C	109.5
O11—P2—C13	108.50 (7)	H15A—C15—H15B	109.5
O12—P2—O11	101.94 (6)	H15A—C15—H15C	109.5
O12—P2—C13	100.62 (7)	H15B—C15—H15C	109.5
O5—P3—O9	112.76 (7)	O8—C16—H16A	110.0
O5—P3—O10	116.32 (7)	O8—C16—H16B	110.0
O5—P3—C12	114.34 (7)	O8—C16—C17	108.68 (15)
O9—P3—C12	108.77 (7)	H16A—C16—H16B	108.3
O10—P3—O9	102.72 (6)	C17—C16—H16A	110.0
O10—P3—C12	100.66 (7)	C17—C16—H16B	110.0
C14—O7—P1	119.89 (10)	C16—C17—H17A	109.5
C16—O8—P1	119.61 (11)	C16—C17—H17B	109.5
C18—O9—P3	120.98 (10)	C16—C17—H17C	109.5
C20—O10—P3	121.18 (11)	H17A—C17—H17B	109.5
C22—O11—P2	123.56 (10)	H17A—C17—H17C	109.5
C24—O12—P2	119.46 (10)	H17B—C17—H17C	109.5
C5—N1—H1	121.2 (14)	O9—C18—H18A	110.2
C8—N1—H1	117.1 (14)	O9—C18—H18B	110.2
C8—N1—C5	121.51 (15)	O9—C18—C19	107.77 (14)
C6—N2—H2	119.9 (16)	H18A—C18—H18B	108.5
C9—N2—H2	117.7 (16)	C19—C18—H18A	110.2
C9—N2—C6	122.08 (14)	C19—C18—H18B	110.2
C7—N3—H3	119.5 (15)	C18—C19—H19A	109.5
C10—N3—H3	117.9 (15)	C18—C19—H19B	109.5
C10—N3—C7	122.40 (15)	C18—C19—H19C	109.5
C2—C1—H1A	109.5 (12)	H19A—C19—H19B	109.5
C2—C1—C3	107.39 (17)	H19A—C19—H19C	109.5
C3—C1—H1A	108.6 (12)	H19B—C19—H19C	109.5
C4—C1—H1A	111.6 (12)	O10—C20—H20A	109.7
C4—C1—C2	110.97 (17)	O10—C20—H20B	109.7
C4—C1—C3	108.61 (16)	O10—C20—C21	109.73 (18)
C2A—C1—H1A	103.4 (12)	H20A—C20—H20B	108.2
C3A—C1—H1A	103.2 (12)	C21—C20—H20A	109.7
C3A—C1—C2A	114.5 (3)	C21—C20—H20B	109.7
C3A—C1—C4A	118.3 (3)	C20—C21—H21A	109.5
C4A—C1—H1A	104.2 (12)	C20—C21—H21B	109.5
C4A—C1—C2A	111.1 (3)	C20—C21—H21C	109.5
N1—C5—H5AA	109.0	H21A—C21—H21B	109.5
N1—C5—H5AB	109.0	H21A—C21—H21C	109.5
N1—C5—H5BC	110.0	H21B—C21—H21C	109.5
N1—C5—H5BD	110.0	O11—C22—H22A	109.9
N1—C5—C2	113.05 (14)	O11—C22—H22B	109.9
N1—C5—C2A	108.5 (2)	O11—C22—C23	108.74 (14)
H5AA—C5—H5AB	107.8	H22A—C22—H22B	108.3
H5BC—C5—H5BD	108.4	C23—C22—H22A	109.9
C2—C5—H5AA	109.0	C23—C22—H22B	109.9
C2—C5—H5AB	109.0	C22—C23—H23A	109.5
C2A—C5—H5BC	110.0	C22—C23—H23B	109.5
C2A—C5—H5BD	110.0	C22—C23—H23C	109.5
N2—C6—H6AA	109.2	H23A—C23—H23B	109.5
N2—C6—H6AB	109.2	H23A—C23—H23C	109.5
N2—C6—H6BC	109.5	H23B—C23—H23C	109.5
N2—C6—H6BD	109.5	O12—C24—H24A	110.2
N2—C6—C3	111.97 (16)	O12—C24—H24B	110.2
N2—C6—C3A	110.5 (2)	O12—C24—C25	107.54 (16)
H6AA—C6—H6AB	107.9	H24A—C24—H24B	108.5
H6BC—C6—H6BD	108.1	C25—C24—H24A	110.2
C3—C6—H6AA	109.2	C25—C24—H24B	110.2
C3—C6—H6AB	109.2	C24—C25—H25A	109.5
C3A—C6—H6BC	109.5	C24—C25—H25B	109.5
C3A—C6—H6BD	109.5	C24—C25—H25C	109.5
N3—C7—H7AA	109.1	H25A—C25—H25B	109.5
N3—C7—H7AB	109.1	H25A—C25—H25C	109.5
N3—C7—H7BC	110.1	H25B—C25—H25C	109.5
N3—C7—H7BD	110.1	C1—C2—C5	112.41 (18)
N3—C7—C4	112.46 (15)	C1—C2—H2A	109.1
N3—C7—C4A	107.8 (2)	C1—C2—H2B	109.1
H7AA—C7—H7AB	107.8	C5—C2—H2A	109.1
H7BC—C7—H7BD	108.5	C5—C2—H2B	109.1
C4—C7—H7AA	109.1	H2A—C2—H2B	107.9
C4—C7—H7AB	109.1	C1—C3—H3A	108.8
C4A—C7—H7BC	110.1	C1—C3—H3B	108.8
C4A—C7—H7BD	110.1	C6—C3—C1	113.63 (17)
O1—C8—N1	123.86 (14)	C6—C3—H3A	108.8
O1—C8—C11	121.44 (14)	C6—C3—H3B	108.8
N1—C8—C11	114.70 (14)	H3A—C3—H3B	107.7
O2—C9—N2	123.68 (15)	C1—C4—C7	114.59 (17)
O2—C9—C12	121.02 (15)	C1—C4—H4A	108.6
N2—C9—C12	115.30 (14)	C1—C4—H4B	108.6
O3—C10—N3	124.25 (15)	C7—C4—H4A	108.6
O3—C10—C13	120.97 (15)	C7—C4—H4B	108.6
N3—C10—C13	114.78 (14)	H4A—C4—H4B	107.6
P1—C11—H11A	109.7	C1—C2A—H2AA	108.8
P1—C11—H11B	109.7	C1—C2A—H2AB	108.8
C8—C11—P1	109.70 (10)	C5—C2A—C1	113.6 (3)
C8—C11—H11A	109.7	C5—C2A—H2AA	108.8
C8—C11—H11B	109.7	C5—C2A—H2AB	108.8
H11A—C11—H11B	108.2	H2AA—C2A—H2AB	107.7
P3—C12—H12A	109.5	C1—C3A—C6	114.5 (3)
P3—C12—H12B	109.5	C1—C3A—H3AA	108.6
C9—C12—P3	110.80 (11)	C1—C3A—H3AB	108.6
C9—C12—H12A	109.5	C6—C3A—H3AA	108.6
C9—C12—H12B	109.5	C6—C3A—H3AB	108.6
H12A—C12—H12B	108.1	H3AA—C3A—H3AB	107.6
P2—C13—H13A	109.6	C1—C4A—H4AA	107.4
P2—C13—H13B	109.6	C1—C4A—H4AB	107.4
C10—C13—P2	110.07 (11)	C7—C4A—C1	119.9 (3)
C10—C13—H13A	109.6	C7—C4A—H4AA	107.4
C10—C13—H13B	109.6	C7—C4A—H4AB	107.4
H13A—C13—H13B	108.2	H4AA—C4A—H4AB	106.9
			
P1—O7—C14—C15	−169.31 (13)	N2—C9—C12—P3	114.58 (14)
P1—O8—C16—C17	131.70 (16)	N3—C7—C4—C1	55.4 (2)
P2—O11—C22—C23	119.26 (14)	N3—C7—C4A—C1	−62.0 (4)
P2—O12—C24—C25	151.86 (17)	N3—C10—C13—P2	126.59 (13)
P3—O9—C18—C19	142.53 (12)	C5—N1—C8—O1	3.3 (2)
P3—O10—C20—C21	−145.69 (17)	C5—N1—C8—C11	−176.51 (13)
O1—C8—C11—P1	−70.88 (17)	C6—N2—C9—O2	2.7 (3)
O2—C9—C12—P3	−65.17 (18)	C6—N2—C9—C12	−177.08 (14)
O3—C10—C13—P2	−53.21 (19)	C7—N3—C10—O3	3.7 (3)
O4—P1—O7—C14	31.54 (14)	C7—N3—C10—C13	−176.07 (14)
O4—P1—O8—C16	52.59 (15)	C8—N1—C5—C2	78.0 (2)
O4—P1—C11—C8	−41.31 (13)	C8—N1—C5—C2A	138.4 (2)
O5—P3—O9—C18	33.23 (14)	C9—N2—C6—C3	87.1 (2)
O5—P3—O10—C20	62.32 (17)	C9—N2—C6—C3A	143.7 (2)
O5—P3—C12—C9	−45.33 (13)	C10—N3—C7—C4	79.6 (2)
O6—P2—O11—C22	27.99 (14)	C10—N3—C7—C4A	132.3 (3)
O6—P2—O12—C24	56.73 (14)	C11—P1—O7—C14	−96.14 (13)
O6—P2—C13—C10	−34.53 (14)	C11—P1—O8—C16	176.58 (13)
O7—P1—O8—C16	−70.93 (14)	C12—P3—O9—C18	−94.69 (13)
O7—P1—C11—C8	85.75 (12)	C12—P3—O10—C20	−173.55 (16)
O8—P1—O7—C14	157.15 (12)	C13—P2—O11—C22	−100.87 (13)
O8—P1—C11—C8	−166.67 (11)	C13—P2—O12—C24	−178.94 (12)
O9—P3—O10—C20	−61.33 (16)	C2—C1—C3—C6	66.6 (2)
O9—P3—C12—C9	81.70 (12)	C2—C1—C4—C7	−174.01 (17)
O10—P3—O9—C18	159.22 (12)	C3—C1—C2—C5	−175.92 (16)
O10—P3—C12—C9	−170.81 (11)	C3—C1—C4—C7	68.2 (2)
O11—P2—O12—C24	−67.24 (13)	C4—C1—C2—C5	65.5 (2)
O11—P2—C13—C10	93.74 (12)	C4—C1—C3—C6	−173.29 (18)
O12—P2—O11—C22	153.50 (12)	C2A—C1—C3A—C6	177.4 (3)
O12—P2—C13—C10	−159.72 (12)	C2A—C1—C4A—C7	−47.4 (5)
N1—C5—C2—C1	61.5 (2)	C3A—C1—C2A—C5	−43.5 (4)
N1—C5—C2A—C1	−69.9 (3)	C3A—C1—C4A—C7	177.1 (3)
N1—C8—C11—P1	108.97 (13)	C4A—C1—C2A—C5	179.2 (3)
N2—C6—C3—C1	62.7 (2)	C4A—C1—C3A—C6	−48.6 (5)
N2—C6—C3A—C1	−60.9 (4)		

### Diethyl {[(5-[2-(diethoxyphosphoryl)acetamido]-3-{2-[2-(diethoxyphosphoryl)acetamido]ethyl}pentyl)carbamoyl]methyl}phosphonate Hydrogen-bond geometry (Å, º)

**Table:**

*D*—H···*A*	*D*—H	H···*A*	*D*···*A*	*D*—H···*A*
N1—H1···O6	0.86 (2)	2.07 (2)	2.9138 (18)	168.8 (19)
N2—H2···O4	0.79 (2)	2.06 (2)	2.8465 (18)	170 (2)
N3—H3···O5	0.82 (2)	2.10 (2)	2.8975 (19)	167 (2)
C11—H11*A*···O6	0.99	2.36	3.2433 (19)	148
C11—H11*B*···O6^i^	0.99	2.48	3.3235 (19)	143
C12—H12*A*···O4	0.99	2.37	3.2476 (19)	148
C12—H12*B*···O2^ii^	0.99	2.35	3.321 (2)	168
C13—H13*A*···O5	0.99	2.37	3.259 (2)	149
C14—H14*A*···O1	0.99	2.56	3.326 (2)	135
C17—H17*B*···O3^iii^	0.98	2.65	3.427 (3)	137
C18—H18*A*···O2	0.99	2.57	3.215 (2)	122
C22—H22*B*···O1^i^	0.99	2.80	3.472 (2)	126
C23—H23*C*···O3	0.98	2.69	3.460 (2)	135
C24—H24*A*···O1^i^	0.99	2.55	3.480 (2)	156
C24—H24*B*···O8	0.99	2.57	3.444 (2)	147
C4*A*—H4*AA*···O2^iv^	0.99	2.39	3.241 (5)	144

Symmetry codes: (i) −*x*+1, −*y*+1, −*z*; (ii) −*x*+1, −*y*+2, −*z*+1; (iii) *x*−1, *y*, *z*; (iv) −*x*+2, −*y*+2, −*z*+1.
